# How to do a grounded theory study: a worked example of a study of dental practices

**DOI:** 10.1186/1471-2288-11-128

**Published:** 2011-09-09

**Authors:** Alexandra Sbaraini, Stacy M Carter, R Wendell Evans, Anthony Blinkhorn

**Affiliations:** 1 Centre for Values, Ethics and the Law in Medicine, University of Sydney, Sydney, New South Wales, Australia; 2 Population Oral Health, Faculty of Dentistry, University of Sydney, Sydney, New South Wales, Australia

**Keywords:** qualitative research, grounded theory, methodology, methods, dental care

## Abstract

**Background:**

Qualitative methodologies are increasingly popular in medical research. Grounded theory is the methodology most-often cited by authors of qualitative studies in medicine, but it has been suggested that many 'grounded theory' studies are not concordant with the methodology. In this paper we provide a worked example of a grounded theory project. Our aim is to provide a model for practice, to connect medical researchers with a useful methodology, and to increase the quality of 'grounded theory' research published in the medical literature.

**Methods:**

We documented a worked example of using grounded theory methodology in practice.

**Results:**

We describe our sampling, data collection, data analysis and interpretation. We explain how these steps were consistent with grounded theory methodology, and show how they related to one another. Grounded theory methodology assisted us to develop a detailed model of the process of adapting preventive protocols into dental practice, and to analyse variation in this process in different dental practices.

**Conclusions:**

By employing grounded theory methodology rigorously, medical researchers can better design and justify their methods, and produce high-quality findings that will be more useful to patients, professionals and the research community.

## Background

Qualitative research is increasingly popular in health and medicine. In recent decades, qualitative researchers in health and medicine have founded specialist journals, such as *Qualitative Health Research*, established 1991, and specialist conferences such as the Qualitative Health Research conference of the International Institute for Qualitative Methodology, established 1994, and the Global Congress for Qualitative Health Research, established 2011 [1-3]. Journals such as the *British Medical Journal *have published series about qualitative methodology (1995 and 2008) [4,5]. Bodies overseeing human research ethics, such as the Canadian Tri-Council Policy Statement: Ethical Conduct for Research Involving Humans, and the Australian National Statement on Ethical Conduct in Human Research [6,7], have included chapters or sections on the ethics of qualitative research. The increasing popularity of qualitative methodologies for medical research has led to an increasing awareness of formal qualitative methodologies. This is particularly so for grounded theory, one of the most-cited qualitative methodologies in medical research [[8], p47].

Grounded theory has a chequered history [9]. Many authors label their work 'grounded theory' but do not follow the basics of the methodology [10,11]. This may be in part because there are few practical examples of grounded theory in use in the literature. To address this problem, we will provide a brief outline of the history and diversity of grounded theory methodology, and a worked example of the methodology in practice. Our aim is to provide a model for practice, to connect medical researchers with a useful methodology, and to increase the quality of 'grounded theory' research published in the medical literature.

### The history, diversity and basic components of 'grounded theory' methodology and method

Founded on the seminal 1967 book 'The Discovery of Grounded Theory' [12], the grounded theory tradition is now diverse and somewhat fractured, existing in four main types, with a fifth emerging. Types one and two are the work of the original authors: Barney Glaser's 'Classic Grounded Theory' [13] and Anselm Strauss and Juliet Corbin's 'Basics of Qualitative Research' [14]. Types three and four are Kathy Charmaz's 'Constructivist Grounded Theory' [15] and Adele Clarke's postmodern Situational Analysis [16]: Charmaz and Clarke were both students of Anselm Strauss. The fifth, emerging variant is 'Dimensional Analysis' [17] which is being developed from the work of Leonard Schaztman, who was a colleague of Strauss and Glaser in the 1960s and 1970s.

There has been some discussion in the literature about what characteristics a grounded theory study must have to be legitimately referred to as 'grounded theory' [18]. The fundamental components of a grounded theory study are set out in Table 1. These components may appear in different combinations in other qualitative studies; a grounded theory study should have all of these. As noted, there are few examples of 'how to do' grounded theory in the literature [18,19]. Those that do exist have focused on Strauss and Corbin's methods [20-25]. An exception is Charmaz's own description of her study of chronic illness [26]; we applied this same variant in our study. In the remainder of this paper, we will show how each of the characteristics of grounded theory methodology worked in our study of dental practices.

**Table 1:** Fundamental components of a grounded theory study

COMPONENT	STAGE	DESCRIPTION	SOURCES
Openness	Throughout the study	Grounded theory methodology emphasises inductive analysis. Deduction is the usual form of analytic thinking in medical research. Deduction moves from the general to the particular: it begins with pre-existing hypotheses or theories, and collects data to test those theories. In contrast, induction moves from the particular to the general: it develops new theories or hypotheses from many observations. Grounded theory particularly emphasises induction. This means that grounded theory studies tend to take a very open approach to the process being studied. The emphasis of a grounded theory study may evolve as it becomes apparent to the researchers what is important to the study participants.	[8] p1-3, 15,16,43- 46 [12] p2-6 [15] p4-21
Analysing immediately	Analysis and data collection	In a grounded theory study, the researchers do not wait until the data are collected before commencing analysis. In a grounded theory study, analysis must commence as soon as possible, and continue in parallel with data collection, to allow *theoretical sampling *(see below).	[8] p12,13, 301 [12] p102 [15] p20
Coding and comparing	Analysis	Data analysis relies on *coding *- a process of breaking data down into much smaller components and labelling those components - and *comparing *- comparing data with data, case with case, event with event, code with code, to understand and explain variation in the data. *Codes *are eventually combined and related to one another - at this stage they are more abstract, and are referred to as *categories *or *concepts*.	[8] p80,81, 265-289 [12] p101-115 [15] p42-71
Memo-writing (sometimes also drawing diagrams)	Analysis	The analyst writes many memos throughout the project. Memos can be about events, cases, categories, or relationships between categories. Memos are used to stimulate and record the analysts' developing thinking, including the *comparisons *made (see above).	[8] p245-264,281, 282,302 [12] p108,112 [15] p72-95
Theoretical sampling	Sampling and data collection	Theoretical sampling is central to grounded theory design. A theoretical sample is informed by *coding, comparison and memo-writing*. Theoretical sampling is designed to serve the developing *theory*. Analysis raises questions, suggests relationships, highlights gaps in the existing data set and reveals what the researchers do not yet know. By carefully selecting *participants *and by modifying the *questions *asked in data collection, the researchers fill gaps, clarify uncertainties, test their interpretations, and build their emerging theory.	[8] p304, 305, 611 [12] p45-77 [15] p96-122
Theoretical saturation	Sampling, data collection and analysis	Qualitative researchers generally seek to reach 'saturation' in their studies. Often this is interpreted as meaning that the researchers are hearing nothing new from participants. In a grounded theory study, theoretical saturation is sought. This is a subtly different form of saturation, in which all of the concepts in the substantive theory being developed are well understood and can be substantiated from the data.	[8] p306, 281,611 [12] p111-113 [15] p114, 115
Production of a substantive theory	Analysis and interpretation	The results of a grounded theory study are expressed as a substantive theory, that is, as a set of concepts that are related to one another in a cohesive whole. As in most science, this theory is considered to be fallible, dependent on context and never completely final.	[8] p14,25 [12] p21-43 [15] p123-150

### Study background

We used grounded theory methodology to investigate social processes in private dental practices in New South Wales (NSW), Australia. This grounded theory study builds on a previous Australian Randomized Controlled Trial (RCT) called the Monitor Dental Practice Program (MPP) [27]. We know that preventive techniques can arrest early tooth decay and thus reduce the need for fillings [28-32]. Unfortunately, most dentists worldwide who encounter early tooth decay continue to drill it out and fill the tooth [33-37]. The MPP tested whether dentists could increase their use of preventive techniques. In the intervention arm, dentists were provided with a set of evidence-based preventive protocols to apply [38]; control practices provided usual care. The MPP protocols used in the RCT guided dentists to systematically apply preventive techniques to prevent new tooth decay and to arrest early stages of tooth decay in their patients, therefore reducing the need for drilling and filling. The protocols focused on (1) primary prevention of new tooth decay (tooth brushing with high concentration fluoride toothpaste and dietary advice) and (2) intensive secondary prevention through professional treatment to arrest tooth decay progress (application of fluoride varnish, supervised monitoring of dental plaque control and clinical outcomes)[38].

As the RCT unfolded, it was discovered that practices in the intervention arm were not implementing the preventive protocols uniformly. Why had the outcomes of these systematically implemented protocols been so different? This question was the starting point for our grounded theory study. We aimed to understand how the protocols had been implemented, including the conditions and consequences of variation in the process. We hoped that such understanding would help us to see how the norms of Australian private dental practice as regards to tooth decay could be moved away from drilling and filling and towards evidence-based preventive care.

#### Designing this grounded theory study

**Figure 1:**
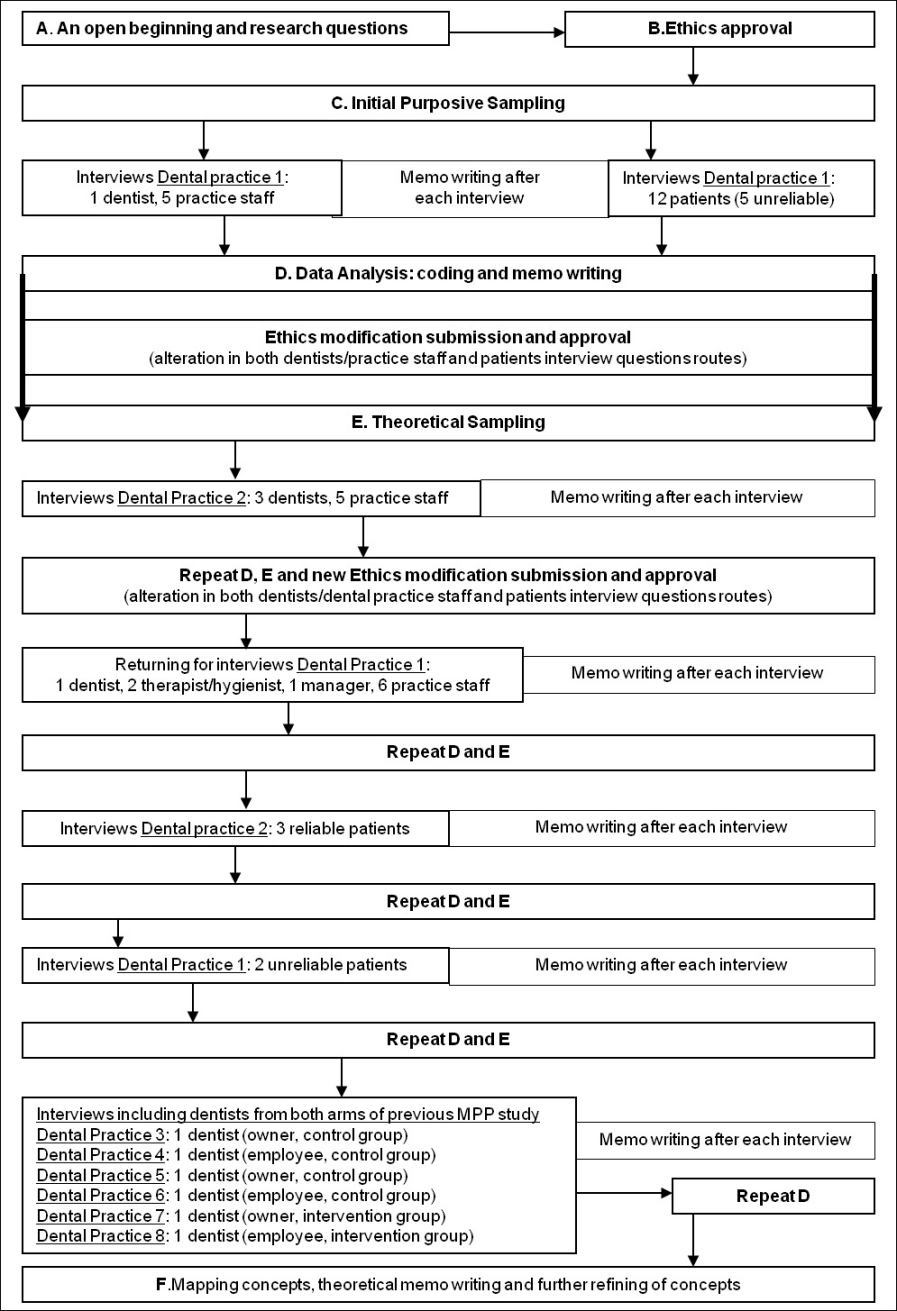
**Study design**. file containing a figure illustrating the study design.

Figure 1 illustrates the steps taken during the project that will be described below from points A to F.

### A. An open beginning and research questions

Grounded theory studies are generally focused on social processes or actions: they ask about *what happens *and *how people interact*. This shows the influence of symbolic interactionism, a social psychological approach focused on the meaning of human actions [39]. Grounded theory studies begin with open questions, and researchers presume that they may know little about the meanings that drive the actions of their participants. Accordingly, we sought to learn from participants how the MPP process worked and how they made sense of it. We wanted to answer a practical social problem: how do dentists persist in drilling and filling early stages of tooth decay, when they could be applying preventive care?

We asked research questions that were open, and focused on social processes. Our initial research questions were:

• What was the process of implementing (or not-implementing) the protocols (from the perspective of dentists, practice staff, and patients)?

• How did this process vary?

### B. Ethics approval and ethical issues

In our experience, medical researchers are often concerned about the ethics oversight process for such a flexible, unpredictable study design. We managed this process as follows. Initial ethics approval was obtained from the Human Research Ethics Committee at the University of Sydney. In our application, we explained grounded theory procedures, in particular the fact that they evolve. In our initial application we provided a long list of possible recruitment strategies and interview questions, as suggested by Charmaz [15]. We indicated that we would make future applications to modify our protocols. We did this as the study progressed - detailed below. Each time we reminded the committee that our study design was intended to evolve with ongoing modifications. Each modification was approved without difficulty. As in any ethical study, we ensured that participation was voluntary, that participants could withdraw at any time, and that confidentiality was protected. All responses were anonymised before analysis, and we took particular care not to reveal potentially identifying details of places, practices or clinicians.

### C. Initial, Purposive Sampling (before theoretical sampling was possible)

Grounded theory studies are characterised by theoretical sampling, but this requires some data to be collected and analysed. Sampling must thus begin purposively, as in any qualitative study. Participants in the previous MPP study provided our population [27]. The MPP included 22 private dental practices in NSW, randomly allocated to either the intervention or control group. With permission of the ethics committee; we sent letters to the participants in the MPP, inviting them to participate in a further qualitative study. From those who agreed, we used the quantitative data from the MPP to select an initial sample.

Then, we selected the practice in which the most dramatic results had been achieved in the MPP study (Dental Practice 1). This was a purposive sampling strategy, to give us the best possible access to the process of *successfully *implementing the protocols. We interviewed all consenting staff who had been involved in the MPP (one dentist, five dental assistants). We then recruited 12 patients who had been enrolled in the MPP, based on their clinically measured risk of developing tooth decay: we selected some patients whose risk status had gotten better, some whose risk had worsened and some whose risk had stayed the same. This purposive sample was designed to provide maximum variation in patients' adoption of preventive dental care.

#### Initial Interviews

One hour in-depth interviews were conducted. The researcher/interviewer (AS) travelled to a rural town in NSW where interviews took place. The initial 18 participants (one dentist, five dental assistants and 12 patients) from Dental Practice 1 were interviewed in places convenient to them such as the dental practice, community centres or the participant's home.

Two initial interview schedules were designed for each group of participants: 1) dentists and dental practice staff and 2) dental patients. Interviews were semi-structured and based loosely on the research questions. The initial questions for dentists and practice staff are in Additional file 1. Interviews were digitally recorded and professionally transcribed. The research location was remote from the researcher's office, thus data collection was divided into two episodes to allow for intermittent data analysis. Dentist and practice staff interviews were done in one week. The researcher wrote memos throughout this week. The researcher then took a month for data analysis in which coding and memo-writing occurred. Then during a return visit, patient interviews were completed, again with memo-writing during the data-collection period.

### D. Data Analysis

#### Coding and the constant comparative method

Coding is essential to the development of a grounded theory [15]. According to Charmaz [[15], p46], 'coding is the pivotal link between collecting data and developing an emergent theory to explain these data. Through coding, you define what is happening in the data and begin to grapple with what it means'. Coding occurs in stages. In initial coding, the researcher generates as many ideas as possible inductively from early data. In focused coding, the researcher pursues a selected set of central codes throughout the entire dataset and the study. This requires decisions about which initial codes are most prevalent or important, and which contribute most to the analysis. In theoretical coding, the researcher refines the final categories in their theory and relates them to one another. Charmaz's method, like Glaser's method [13], captures actions or processes by using gerunds as codes (verbs ending in 'ing'); Charmaz also emphasises coding quickly, and keeping the codes as similar to the data as possible.

We developed our coding systems individually and through team meetings and discussions.

We have provided a worked example of coding in Table 2. Gerunds emphasise actions and processes. Initial coding identifies many different processes. After the first few interviews, we had a large amount of data and many initial codes. This included a group of codes that captured how dentists sought out evidence when they were exposed to a complex clinical case, a new product or technique. Because this process seemed central to their practice, and because it was talked about often, we decided that seeking out evidence should become a focused code. By comparing codes against codes and data against data, we distinguished the category of "seeking out evidence" from other focused codes, such as "gathering and comparing peers' evidence to reach a conclusion", and we understood the relationships between them. Using this constant comparative method (see Table 1), we produced a theoretical code: "making sense of evidence and constructing knowledge". This code captured the social process that dentists went through when faced with new information or a practice challenge. This theoretical code will be the focus of a future paper.

**Table 2:** Coding process

Raw data	Initial coding	Focused coding	*Theoretical coding*
Q. What did you take into account when you decided to buy this new technology? What did we... we looked at cost, we looked at reliability and we sort of, we compared a few different types, talked to some people that had them. Q. When you say you talked to some people who were they? Some dental colleagues. There's a couple of internet sites that we talked to some people... people had tried out some that didn't work very well. Q. So in terms of materials either preventive materials or restorative materials; what do you take in account when you decide which one to adopt? Well, that's a good question. I don't know. I suppose we [laughs] look at reliability. I suppose I've been looking at literature involved in it so I quite like my own little research about that, because I don't really trust the research that comes with the product and once again what other dentists are using and what they've been using and they're happy with. I'm finding the internet, some of those internet forums are actually quite good for new products.	Deciding to buy based on cost, reliability Talking to dental colleagues on internet sites Comparing their experiences Looking at literature Doing my own little research Not trusting research that comes with commercial products Talking to other dentists about their experiences	**Seeking out evidence** **Gathering and comparing peers' evidence to reach a conclusion**	*The process of making sense of evidence and construction of knowledge*

### Memo-writing

Throughout the study, we wrote extensive case-based memos and conceptual memos. After each interview, the interviewer/researcher (AS) wrote a case-based memo reflecting on what she learned from that interview. They contained the interviewer's impressions about the participants' experiences, and the interviewer's reactions; they were also used to systematically question some of our pre-existing ideas in relation to what had been said in the interview. Table 3 illustrates one of those memos. After a few interviews, the interviewer/researcher also began making and recording comparisons among these memos.

**Table 3:** Case-based memo

**Memo written after interviewing a practice manager**

This was quite an eye opening interview in the sense that the practice manager was very direct, practical and open. In his accounts, the bottom line is that this preventive program is not profitable; dentists will do it for giving back to the community, not to earn money from it. I am so glad we had this interview; otherwise I am not sure if someone would be so up front about it. So, my question really is, is that the reason why dentists have not adopted it in other practices? And what about other patients who come here, who are not enrolled in the research program, does the dentist-in-charge treat them all as being part of the program or it was just an impression from the interview and what I saw here during my time in the practice... or will the dentist continue doing it in the next future?
I definitely learned that dentistry in private practice is a business, at the end of the day a target has to be achieved, and the dentist is driven by it. During the dentist's interview, there was a story about new patients being referred to the practice because the way they were treating patients now; but right now I am just not sure; I really need to check that... need to go back and ask the dentist about it, were there any referrals or not? Because this would create new revenue for the practice and the practice manager would surely be happy about it. On the other hand, it is interesting that the practice manager thinks that having a hygienist who was employed few months ago is the way to adopt the preventive program; she should implement it, freeing the dentist to do more complex work. But in reality, when I interviewed the hygienist I learned that she does not want to change to adopt the program, she is really focused on what she has been doing for a while and trust her experience a lot! So I guess, the dentist in charge might be going through a new changing process, different from what happen when the MPP protocols were first tried in this practice; this is another point to check on the next interview with the dentist. I just have this feeling that somehow the new staff (hygienist) is really important for this practice to regain and maintain profit throughout the adoption of preventive protocols but there are some personality clashes happening along the way.

We also wrote conceptual memos about the initial codes and focused codes being developed, as described by Charmaz [15]. We used these memos to record our thinking about the meaning of codes and to record our thinking about how and when processes occurred, how they changed, and what their consequences were. In these memos, we made comparisons between data, cases and codes in order to find similarities and differences, and raised questions to be answered in continuing interviews. Table 4 illustrates a conceptual memo.

**Table 4:** Conceptual memo

**Believing + Embracing + Developing = Adapting?**

In these dental practices the adaptation to preventive protocols was all about believing in this new approach to manage dental caries and in themselves as professionals. New concepts were embraced and slowly incorporated into practice. Embracing new concepts/paradigms/systems and abandoning old ones was quite evident during this process (old concepts = dentistry restorative model; new concepts = non-surgical approach). This evolving process involved feelings such as anxiety, doubt, determination, confidence, and reassurance. The modification of practices was possible when dentists-in-charge felt that perhaps there was something else that would be worth doing; something that might be a little different from what was done so far. The responsibility to offer the best available treatment might have triggered this reasoning. However, there are other factors that play an important role during this process such as dentist's personal features, preconceived notions, dental practice environment, and how dentists combine patients' needs and expectations while making treatment decisions. Finding the balance between preventive non-surgical treatment (curing of disease) and restorative treatment (making up for lost tissues) is an every moment challenge in a profitable dental practice. Regaining profit, reassessing team work and surgery logistics, and mastering the scheduling art to maximize financial and clinical outcomes were important practical issues tackled in some of these practices during this process.
These participants talked about learning and adapting new concepts to their practices and finally never going back the way it was before. This process brought positive changes to participants' daily activities. Empowerment of practice staff made them start to enjoy more their daily work (they were recognized by patients as someone who was truly interested in delivering the best treatment for them). Team members realized that there were many benefits to patients and to staff members in implementing this program, such as, professional development, offering the best care for each patient and job satisfaction.

At the end of our data collection and analysis from Dental Practice 1, we had developed a tentative model of the process of implementing the protocols, from the perspective of dentists, dental practice staff and patients. This was expressed in both diagrams and memos, was built around a core set of focused codes, and illustrated relationships between them.

### E. Theoretical sampling, ongoing data analysis and alteration of interview route

We have already described our initial purposive sampling. After our initial data collection and analysis, we used theoretical sampling (see Table 1) to determine who to sample next and what questions to ask during interviews. We submitted Ethics Modification applications for changes in our question routes, and had no difficulty with approval. We will describe how the interview questions for dentists and dental practice staff evolved, and how we selected new participants to allow development of our substantive theory. The patients' interview schedule and theoretical sampling followed similar procedures.

#### Evolution of theoretical sampling and interview questions

We now had a detailed provisional model of the successful process implemented in Dental Practice 1. Important core focused codes were identified, including practical/financial, historical and philosophical dimensions of the process. However, we did not yet understand how the process might vary or go wrong, as implementation in the first practice we studied had been described as seamless and beneficial for everyone. Because our aim was to understand the process of implementing the protocols, including the conditions and consequences of variation in the process, we needed to understand how implementation might fail. For this reason, we theoretically sampled participants from Dental Practice 2, where uptake of the MPP protocols had been very limited according to data from the RCT trial.

We also changed our interview questions based on the analysis we had already done (see Additional file 2). In our analysis of data from Dental Practice 1, we had learned that "effectiveness" of treatments and "evidence" both had a range of meanings. We also learned that new technologies - in particular digital x-rays and intra-oral cameras - had been unexpectedly important to the process of implementing the protocols. For this reason, we added new questions for the interviews in Dental Practice 2 to directly investigate "effectiveness", "evidence" and how dentists took up new technologies in their practice.

Then, in Dental Practice 2 we learned more about the barriers dentists and practice staff encountered during the process of implementing the MPP protocols. We confirmed and enriched our understanding of dentists' processes for adopting technology and producing knowledge, dealing with complex cases and we further clarified the concept of evidence. However there was a new, important, unexpected finding in Dental Practice 2. Dentists talked about "unreliable" patients - that is, patients who were too unreliable to have preventive dental care offered to them. This seemed to be a potentially important explanation for non-implementation of the protocols. We modified our interview schedule again to include questions about this concept (see Additional file 3) leading to another round of ethics approvals. We also returned to Practice 1 to ask participants about the idea of an "unreliable" patient.

Dentists' construction of the "unreliable" patient during interviews also prompted us to theoretically sample for "unreliable" and "reliable" patients in the following round of patients' interviews. The patient question route was also modified by the analysis of the dentists' and practice staff data. We wanted to compare dentists' perspectives with the perspectives of the patients themselves. Dentists were asked to select "reliable" and "unreliable" patients to be interviewed. Patients were asked questions about what kind of services dentists should provide and what patients valued when coming to the dentist. We found that these patients (10 reliable and 7 unreliable) talked in very similar ways about dental care. This finding suggested to us that some deeply-held assumptions within the dental profession may not be shared by dental patients.

At this point, we decided to theoretically sample dental practices from the non-intervention arm of the MPP study. This is an example of the 'openness' of a grounded theory study potentially subtly shifting the focus of the study. Our analysis had shifted our focus: rather than simply studying the process of implementing the evidence-based preventive protocols, we were studying the process of doing prevention in private dental practice. All participants seemed to be revealing deeply held perspectives shared in the dental profession, whether or not they were providing dental care as outlined in the MPP protocols. So, by sampling dentists from both intervention and control group from the previous MPP study, we aimed to confirm or disconfirm the broader reach of our emerging theory and to complete inductive development of key concepts. Theoretical sampling added 12 face to face interviews and 10 telephone interviews to the data. A total of 40 participants between the ages of 18 and 65 were recruited. Telephone interviews were of comparable length, content and quality to face to face interviews, as reported elsewhere in the literature [40].

### F. Mapping concepts, theoretical memo writing and further refining of concepts

After theoretical sampling, we could begin coding theoretically. We fleshed out each major focused code, examining the situations in which they appeared, when they changed and the relationship among them. At time of writing, we have reached theoretical saturation (see Table 1). We have been able to determine this in several ways. As we have become increasingly certain about our central focused codes, we have re-examined the data to find all available insights regarding those codes. We have drawn diagrams and written memos. We have looked rigorously for events or accounts not explained by the emerging theory so as to develop it further to explain all of the data. Our theory, which is expressed as a set of concepts that are related to one another in a cohesive way, now accounts adequately for all the data we have collected. We have presented the developing theory to specialist dental audiences and to the participants, and have found that it was accepted by and resonated with these audiences.

We have used these procedures to construct a detailed, multi-faceted model of the process of incorporating prevention into private general dental practice. This model includes relationships among concepts, consequences of the process, and variations in the process. A concrete example of one of our final key concepts is the process of "adapting to" prevention. More commonly in the literature writers speak of adopting, implementing or translating evidence-based preventive protocols into practice. Through our analysis, we concluded that what was required was 'adapting to' those protocols in practice. Some dental practices underwent a slow process of adapting evidence-based guidance to their existing practice logistics. Successful adaptation was contingent upon whether (1) the dentist-in-charge brought the whole dental team together - including other dentists - and got everyone interested and actively participating during preventive activities; (2) whether the physical environment of the practice was re-organised around preventive activities, (3) whether the dental team was able to devise new and efficient routines to accommodate preventive activities, and (4) whether the fee schedule was amended to cover the delivery of preventive services, which hitherto was considered as "unproductive time".

Adaptation occurred over time and involved practical, historical and philosophical aspects of dental care. Participants transitioned from their initial state - selling restorative care - through an intermediary stage - learning by doing and educating patients about the importance of preventive care - and finally to a stage where they were offering patients more than just restorative care. These are examples of ways in which participants did not simply adopt protocols in a simple way, but needed to adapt the protocols and their own routines as they moved toward more preventive practice.

#### The quality of this grounded theory study

There are a number of important assurances of quality in keeping with grounded theory procedures and general principles of qualitative research. The following points describe what was crucial for this study to achieve quality.

#### During data collection

1. All interviews were digitally recorded, professionally transcribed in detail and the transcripts checked against the recordings.

2. We analysed the interview transcripts as soon as possible after each round of interviews in each dental practice sampled as shown on Figure 1. This allowed the process of theoretical sampling to occur.

3. Writing case-based memos right after each interview while being in the field allowed the researcher/interviewer to capture initial ideas and make comparisons between participants' accounts. These memos assisted the researcher to make comparison among her reflections, which enriched data analysis and guided further data collection.

4. Having the opportunity to contact participants after interviews to clarify concepts and to interview some participants more than once contributed to the refinement of theoretical concepts, thus forming part of theoretical sampling.

5. The decision to include phone interviews due to participants' preference worked very well in this study. Phone interviews had similar length and depth compared to the face to face interviews, but allowed for a greater range of participation.

#### During data analysis

1. Detailed analysis records were kept; which made it possible to write this explanatory paper.

2. The use of the constant comparative method enabled the analysis to produce not just a description but a model, in which more abstract concepts were related and a social process was explained.

3. All researchers supported analysis activities; a regular meeting of the research team was convened to discuss and contextualize emerging interpretations, introducing a wide range of disciplinary perspectives.

#### Answering our research questions

We developed a detailed model of the process of adapting preventive protocols into dental practice, and analysed the variation in this process in different dental practices. Transferring evidence-based preventive protocols into these dental practices entailed a slow process of adapting the evidence to the existing practices logistics. Important practical, philosophical and historical elements as well as barriers and facilitators were present during a complex adaptation process. Time was needed to allow dentists and practice staff to go through this process of slowly adapting their practices to this new way of working. Patients also needed time to incorporate home care activities and more frequent visits to dentists into their daily routines. Despite being able to adapt or not, all dentists trusted the concrete clinical evidence that they have produced, that is, seeing results in their patients mouths made them believe in a specific treatment approach.

## Concluding remarks

This paper provides a detailed explanation of how a study evolved using grounded theory methodology (GTM), one of the most commonly used methodologies in qualitative health and medical research [[8], p47]. In 2007, Bryant and Charmaz argued:

'Use of GTM, at least as much as any other research method, only develops with experience. Hence the failure of all those attempts to provide clear, mechanistic rules for GTM: there is no 'GTM for dummies'. GTM is based around heuristics and guidelines rather than rules and prescriptions. Moreover, researchers need to be familiar with GTM, in all its major forms, in order to be able to understand how they might adapt it in use or revise it into new forms and variations.' [[8], p17].

Our detailed explanation of our experience in this grounded theory study is intended to provide, vicariously, the kind of 'experience' that might help other qualitative researchers in medicine and health to apply and benefit from grounded theory methodology in their studies. We hope that our explanation will assist others to avoid using grounded theory as an 'approving bumper sticker' [10], and instead use it as a resource that can greatly improve the quality and outcome of a qualitative study.

## Abbreviations

GTM: grounded theory methods; MPP: Monitor Dental Practice Program; NSW: New South Wales; RCT: Randomized Controlled Trial.

## Competing interests

The authors declare that they have no competing interests.

## Authors' contributions

All authors have made substantial contributions to conception and design of this study. AS carried out data collection, analysis, and interpretation of data. SMC made substantial contribution during data collection, analysis and data interpretation. AS, SMC, RWE, and AB have been involved in drafting the manuscript and revising it critically for important intellectual content. All authors read and approved the final manuscript.

## Pre-publication history

The pre-publication history for this paper can be accessed here:

http://www.biomedcentral.com/1471-2288/11/128/prepub

## Supplementary Material

Additional file 1 **Initial interview schedule for dentists and dental practice staff**. file containing initial interview schedule for dentists and dental practice staff. Click here for file

Additional file 2 **Questions added to the initial interview schedule for dentists and dental practice staff**. file containing questions added to the initial interview schedule Click here for file

Additional file 3 **Questions added to the modified interview schedule for dentists and dental practice staff**. file containing questions added to the modified interview schedule Click here for file
