# Supplementary material for: How to do a grounded theory study: a worked example of a study of dental practices
Source: BMC Med Res Methodol. 2011 Sep 9;11:128. doi: 10.1186/1471-2288-11-128 (PMC3184112; doi:10.1186/1471-2288-11-128)
Supplement: Additional file 1 — Initial interview schedule for dentists and dental practice staff. file containing initial interview schedule for dentists and dental practice staff. [file 1471-2288-11-128-S1.DOC]

**Additional file 1**

**Initial interview schedule for dentists and dental practice staff**

- What do you think is the place of prevention in dental practice?
- Tell me about your experience of implementing this preventive approach to manage tooth decay. *
- Take me through your process of interaction with patients regarding the implementation of the preventive protocols.
  - - At what stage did you get involved?
    - Explain the process (case example)
    - What was your specific role?
- When you look back on the implementation process, are there any other events that stand out in your mind? How did the event affect what happened? How did you respond to it?
- How easily were you able to implement the preventive protocols within this practice?
  - - How did all staff working in the practice respond to the implementation? What were some of the issues?
    - What positive changes have occurred in this dental practice since the program was implemented?
    - What negative changes, if any, have occurred in this dental practice since the program was implemented?
- Given your experiences, do you have any suggestions as to how we could better implement a program such as this, both within your practice and to the wider dental community?
- Is there something else you think I should know to understand this implementation process?*
- Is there something you would like to ask me?*

* Source of questions Charmaz [15, p31].
