# Supplementary material for: How to do a grounded theory study: a worked example of a study of dental practices
Source: BMC Med Res Methodol. 2011 Sep 9;11:128. doi: 10.1186/1471-2288-11-128 (PMC3184112; doi:10.1186/1471-2288-11-128)
Supplement: Additional file 2 — Questions added to the initial interview schedule for dentists and dental practice staff. file containing questions added to the initial interview schedule [file 1471-2288-11-128-S2.DOC]

**Additional file 2**

**Questions added to the initial interview schedule for dentists and dental practice staff**

- What would a typical day in this surgery be like? What do you spend most of your time doing?
- Can you think back to a new treatment or technology that you have adopted in your

surgery recently?

- What did you take into account when you decided to adopt it in your

regular practice?

- What did influence your decision to adopt it?
- If you think now about a typical patient, when you are making a decision about how to

treat a patient, what are the things that you would normally consider?

- From your perspective, what are the priorities of this dental practice?
- What are your priorities as a dentist/dental hygienist/dental therapist/dental practice staff?
- There is a lot of talk about preventive dentistry. How do you feel about the idea of

preventive dental practice?

- What are the things that you consider when deciding to intervene either by drilling

and filling or by starting a preventive program with a patient?

- What do you think a dentist/dental hygienist/dental therapist role should be in a practice?
- In an ideal world, what do you think a dentist/dental hygienist/dental therapist

should be able to do for their patients?

- To what extent is it possible to achieve this in the real world?
