# Supplementary material for: How to do a grounded theory study: a worked example of a study of dental practices
Source: BMC Med Res Methodol. 2011 Sep 9;11:128. doi: 10.1186/1471-2288-11-128 (PMC3184112; doi:10.1186/1471-2288-11-128)
Supplement: Additional file 3 — Questions added to the modified interview schedule for dentists and dental practice staff. file containing questions added to the modified interview schedule [file 1471-2288-11-128-S3.DOC]

**Additional file 3**

**Questions added to the modified interview schedule for dentists and dental practice staff**

- Have you ever made a big change in the way you practice dentistry or to the services you provide? Could you tell me about that change?
- In academic dentistry, we often say that the ideal would be to offer preventive treatment to everyone. How possible is this in the real world? [Probe: are there patients who are ideal for prevention and patients who are not ideal?]
- What sorts of preventive services are offered to patients in this practice?
- Think back to when you first agreed to participate in the MPP: what led you to agree to participate?
- Have any aspects of the MPP carried over into your everyday practice now? [If yes, probe why those aspects; if not probe can you tell me about that]
- Think about the MPP preventive protocols. What would it take to make it possible for you to practice like that all the time? [Probe: whether patients will come back; whether the practice can survive financially]
- How likely is it that you would ever practice like that all of the time? [Probe for more info]
- How do you weigh up the financial aspects of practice and the clinical aspects of practice?
- Now I want you to think back to the last time that you decided to do a filling.
- Could you tell me about that patient?
- What was it about that patient that you had to fill the tooth?
- And now could you think of the most recent time when you saw a patient that you decided not to do a filling but to do intensive preventive care to manage their tooth decay.
- Could you tell me how and why you made that decision?
